# Gender Dysphoria: Bioethical Aspects of Medical Treatment

**DOI:** 10.1155/2018/9652305

**Published:** 2018-06-13

**Authors:** Marta R. Bizic, Milos Jeftovic, Slavica Pusica, Borko Stojanovic, Dragana Duisin, Svetlana Vujovic, Vojin Rakic, Miroslav L. Djordjevic

**Affiliations:** ^1^Belgrade Center for Genital Reconstructive Surgery, Serbia; ^2^University Children's Hospital, Belgrade, Serbia; ^3^School of Medicine, University of Belgrade, Serbia; ^4^Center for the Study of Bioethics, University of Belgrade, Serbia

## Abstract

Gender affirmation surgery remains one of the greatest challenges in transgender medicine. In recent years, there have been continuous discussions on bioethical aspects in the treatment of persons with gender dysphoria. Gender reassignment is a difficult process, including not only hormonal treatment with possible surgery but also social discrimination and stigma. There is a great variety between countries in specified tasks involved in gender reassignment, and a complex combination of medical treatment and legal paperwork is required in most cases. The most frequent bioethical questions in transgender medicine pertain to the optimal treatment of adolescents, sterilization as a requirement for legal recognition, role of fertility and parenthood, and regret after gender reassignment. We review the recent literature with respect to any new information on bioethical aspects related to medical treatment of people with gender dysphoria.

## 1. Introduction

Gender dysphoria (GD) represents a condition where a person's gender assigned at birth and the gender with which they identify themselves are incongruent. Hence, these individuals can be very uncomfortable with their biological sex, primary and secondary sex characteristics, and social gender roles and they experience various levels of distress. Presence of public figures who are openly transgender, their appearance in mainstream media, and political and social climate lead to more individuals coming out in the open as to their state. Prevalence rate cannot be correctly estimated considering that people are still hesitant to come forward to health centers. According to DSM-5, the prevalence of gender dysphoria is 0.005-0.014% for adult natal males and 0.002-0.003% for adult natal females [1].

In accordance with their wishes, individuals with this condition can choose the direction in which their transition will proceed. To take the edge off their state, one can choose to go through a social transition. The social transition includes using a different name, pronouns, transformation of physical appearance, use of suitable bathrooms, and taking social roles of the affirmed gender. A more radical approach is the medical transition that includes hormonal and surgical treatment. Medical treatment requires a team of experienced experts, and it usually includes mental health professionals, endocrinologists, and surgeons. Psychiatric assessment is the first step and is very complex because it is necessary to exclude other conditions that might mimic gender dysphoria. The next step is hormonal treatment, under the care of an endocrinologist, which is then followed by “a real-life trial.” Some individuals decide to stop here, while others continue to gender-affirming surgery (GAS). The seventh edition of the Standards of Care of the World Professional Association of Transgender Health (WPATH) offers flexible guidelines for the treatment of people experiencing gender dysphoria and describes the criteria for surgical treatment [2]. Patients undergoing GAS of their choice are required to provide two recommendation letters from certified psychiatrists and a gender specialist, as well as a confirmation of having been on hormonal therapy prescribed by an endocrinologist for a period of a minimum of one year. Gender affirmation surgery refers to all surgical procedures that a patient wishes to undergo in an attempt to become as similar as possible to the desired gender.

Treatment of gender dysphoria always raised numerous ethical issues, and with rapid acknowledgment and recent achievements, new complex issues in medical management have emerged. With unknown etiology and questionable definition (mental/medical illness, social construct, and variation of sex) who can decide, with 100% certainty, what treatment is in the best interest of a particular patient? The most prominent challenges and ethical questions pertain to the treatment of underage individuals, fertility after GAS, and possibility of regret after GAS. Main ethical principles are autonomy, beneficence, nonmaleficence, and informed consent. The individual must have autonomy of thought and intention when making decisions about medical treatment. This is an especially sensitive field in treatment of gender dysphoria, because sometimes the individual's desires, hopes, and expectations might not correlate with reality. Experts must be very straightforward regarding specific possibilities, risks, and benefits of medical treatment, especially considering that the last step in medical transition, GAS, is irreversible. Beneficence implies doing only good, only what is in the patient's best interest. However, some may consider that surgical alteration of healthy organs, in case of GAS, is not in line with this principle. Nonmaleficence must ensure that the treatment does not harm the individual in an emotional, social, or physical sense. Always keeping these principles in mind, WPATH Standards of Care and criteria for diagnosis might not be enough to be ascertain that we are doing the right thing. Although it may seem that an individual fulfills all these criteria on paper, sometimes we can observe their personal disadvantages, youth, impairment, or desperation. It seems that, even with the reassurance and recommendation from a mental health professional, ethical unease cannot be entirely erased because treatment guidelines have preceded the answers to vitally relevant questions [3, 4].

## 2. Transgender Youth

Children represent a small number of individuals with gender dysphoria and in only 10-20% of the children, gender dysphoria will continue to manifest in adolescence [5]. However, psychological therapy and support are highly recommended; while such services are now far more widely available, they are still insufficient to provide for complete wellbeing of these patients. Inadequate management of children with persistent gender dysphoria can lead to isolation, feeling of self-hatred, and suicidal ideas and attempts. Also, “passing through the wrong puberty” can have serious consequences for these individuals. Viable treatment options vary from fully reversible treatment, such as puberty-suppressing gonadotropin-releasing hormone analogues (GnRH) to partly reversible treatment, gonadal steroid treatment, as well as irreversible treatment, such as surgical removal of genitalia and reconstruction of new ones according to the desired gender. Surgery includes bilateral mastectomy with chest reconstruction, hysterectomy with oophorectomy followed by either metoidioplasty or phalloplasty for trans-male individuals, and bilateral orchiectomy with penectomy followed by vulvoplasty and vaginoplasty in trans-female individuals [6].

Pubertal suppression is implemented using GnRH analogues at Tanner 2 or 3 stage of puberty. Hypothalamus produces GnRH at low levels in prepubertal children. Levels become cyclical during puberty, leading to the production of luteinizing hormone (LH) and follicle stimulating hormone (FSH) by the anterior pituitary. LH and FSH stimulate ovaries and testicles to produce sex hormones, estrogen and testosterone, which are responsible for stimulating the growth of genitalia. Also, they lead to the development of breasts, voice deepening, menstrual cycle, and so forth, which transgender youth can find particularly tough to handle [7].

There are only a few reports related to the use of GnRH analogues in transgender youth. De Vries et al. were the first to introduce the concept and research on the use of puberty blockers for treatment of transgender youth. The main idea behind the suppression of endogenous puberty was to decrease distress by preventing the development of “noncongruent” secondary sexual characteristics. This would give young individuals more time to get accustomed to their situation and to better explore their gender. In the examined group, all of 70 eligible candidates showed improved mental health and general functioning. Authors concluded that the treatment was fully reversible, which was one of its main advantages [8]. Despite the positive outcomes in puberty suppression, many experts still have concerns and resist the implementation of this treatment in their regular practice. Viner et al. proposed that GnRH therapy can be physically damaging for teenagers and can lead to unfavorable psychological consequences [9]. Olson-Kennedy et al. also recognized these dilemmas, stating that available data on puberty suppression was limited and many questions remained unanswered [10]. One of the main reasons against this treatment is that going through puberty may help the individual to become congruent with their biological sex, meaning that their GD would not persist into adolescence. Results from Steensma et al. showed that majority of children developed homosexual orientation after completion of the GnRH treatment [11]. As for potential consequences, Hembree recently reported no long-term consequences in follow-up studies of GnRH treatment [12].

Finally, the decision about implementing GnRH treatment is very difficult and cannot be made without ethical dilemmas. Both opponents and advocates of pubertal suppression are guided by the same ethical principles, beneficence, nonmaleficence, and autonomy, but have different views on where these principles lead. A unique and clear overview is necessary, and, to this day, it has not yet been elaborated. Considering that GnRH treatment is relatively new and controversial, additional qualitative research and empirical studies are necessary for appropriate bioethical definitions.

Transgender persons require safe and effective hormonal support to develop the physical characteristics that affirm their gender identity. The main indications for the beginning of hormonal therapy are confirmed persistence of gender dysphoria and adequate mental capacity to give informed consent and accept this partially irreversible treatment. According to the most recent Endocrine Society guidelines, most adolescents develop this capacity by the age of 16 [12]. Also, Hembree et al. recognized some compelling reasons to initiate sex hormonal therapy before 16, but there is little data published on the experiences with this treatment prior to 14 years of age [12]. The main goals of cross-sex hormonal therapy are suppression of endogenous sex hormone secretion, determined by the person's genetic/gonadal sex, and maintaining sex hormone levels within the normal range for the person's affirmed gender. This therapy harmonizes the external appearance with affirmed gender, leading to, in transgender men, male-sounding voice, different fat distribution, increase in muscle mass and, in transgender women, breast growth, decreased facial and body hair, more feminine fat redistribution, and decreased muscle mass [12].

Many studies demonstrated long-term safety and high efficiency of hormonal therapy in transgender adults. For trans-women, Asscheman et al. emphasized a warning to a side effect of particular concern, estrogen-induced hypercoagulability and subsequent venous thromboembolism. Hembree addressed some potential adverse physical effects of testosterone treatment, such as polycythemia vera and dyslipidemia, in transgender men. Generally, a majority of the authors concluded that this therapy was safe, with necessary follow-up for potential complications [12–14]. However, only a few studies looked into the impact of cross-sex hormonal therapy on transgender youth. Jarin et al. performed a retrospective study on 116 adolescents aged 14–25 years with gender dysphoria and have reported minimal impact of hormone treatment. In trans-men, the only findings were an increase in hemoglobin, hematocrit, and body mass index with lowering of high-density lipoprotein levels; in trans-women, only lower testosterone and alanine aminotransferase (ALT) were reported [15]. Olson-Kennedy et al., in their prospective study, found several statistically significant changes in mean values of physiological parameters over time but of no consequence to clinical safety concerns [16]. In both studies, the authors indicated that this cross-sex hormonal therapy is safe for transgender youth over a period of approximately two years. However, the strongest argument against cross-sex therapy lies in the lack of knowledge of its long-term effects, which means that more studies and follow-up information are necessary. One of the questions is a possibility for cross-sex hormonal therapy in individuals below 16 years of age. The authors of the latest guidelines of the Endocrine Society recognized this possibility but only on a “case by case” principle, meaning that age does not always accurately reflect one's readiness for medical interventions. Also, some experts noticed that a clear majority of children on GnRH therapy will decide to pursue cross-sex hormonal therapy. Only a few side effects of using GnRH were observed, such as decreased bone density [17].

Based on bioethical principles, children usually do not have the power to make legal decisions and actions at the initiation of cross-hormonal therapy. Nevertheless, their judgment and opinions should not be disregarded. Cross-sex therapy primarily helps individuals with GD to harmonize their external appearance with their experienced gender. In this case, proper education of the patient and pointing out advantages and shortcomings of such treatment are of crucial importance. Following the principle of beneficence, clinicians are always obliged to help the person and to follow the prescribed hormonal treatment, since there are no better options at this moment. Patients who are denied treatment can develop serious psychological consequences. Generally, the transgender population is at higher risk of self-harm and suicide [18]. A more individualized approach, as in the “case by case” system, will ensure that a right decision is made in accordance with the patient's maturity, age, and judgment.

Gender affirmation surgery is the last step in the medical transition. It is considered to be irreversible and is technically demanding to perform, even for experienced surgeons. According to WPATH Standards of Care, a criterion for eligibility for GAS is “reached legal age of maturity in a given country.” Presumably, the threshold is 18 years of age in most countries [19]. The increasing usage of puberty blockers and pushing the limits for the start of the cross-sex hormone therapy lead to further problems and dilemmas. With these developments, it was only a matter of time before the issue of GAS in minors would arise. Viewpoints are different and vary between the beneficence principle embodied in the motto “doing nothing is doing harm” and the nonmaleficence variation of “the treatment plan that involves less extensive surgery or none at all,” reported by Cohen-Kettenis and Holman, respectively [20, 21].

Changing the legislation for hormonal therapy without GAS increases the gap between the two medical procedures and postpones the desired outcome of the transition. During this interim period, someone living with atypical genitalia can easily be exposed in public and lose control over something that used to be very private [22]. Transgender community is more often targeted by bullying and has higher rates of suicide. Leaving these patients to wait for the final stage in their transition can have an impact on their social and psychological state. Goffman's theory of stigma postulates that the transitioning adolescents must prove their affirmed gender to others [23]. If others question the individual's gender identity, including the presence of gender-congruent genitals, he or she fails to manage the stigma and becomes “discredited.” In addition, postponing romantic relationships and dating until the age of 18 can also lead to psychological struggles and challenges.

On the other hand, the main “technical” issue in case of children treated with puberty blockers lies in their undeveloped genitalia. Thus, the GAS will be more troublesome, especially in case of penile inversion vaginoplasty. Some authors reported autologous skin grafting from donor site or use of bowel segments as viable solutions for this issue [24, 25]. However, the main concern is the possibility of regret after the GAS. As already mentioned in Introduction, GD does not persist through adolescence in the vast majority of children. The results of GAS in transgender minors and their possible regret are a great cause of concern and a huge responsibility for medical professionals [26]. The dilemmas remain: is it better to suffer the consequences of GD or GAS? Are children or teenagers mature enough to make these kinds of decisions? Further research and data are necessary to resolve these crucial dilemmas.

## 3. Fertility

Treatment of GD enables the individuals to continue their life in their affirmed gender. For some transgender individuals, this implies the same as for cisgender persons, marriage or/and children. Members of the transgender population have the same desire for offspring, for the same reasons as the cisgender population, and fertility presents one of the most delicate issues. Infertility in trans-women is caused by orchiectomy as a part of the GAS. Conversely, hysterectomy and oophorectomy eliminate the chance of pregnancy in trans-men. Cross-sex hormonal therapy also has an impact on fertility, but such treatment is not a definitive cause of infertility, due to the possibility of reversal. Three decades ago, Payer described that estrogen in trans-women leads to the reduction of testicular volume and has a strong suppressive effect on sperm motility and density [27]. Testosterone therapy for trans-men leads to reversible amenorrhea according to Van Den Broecke's study in 2001 [28]. Patients are usually at full reproductive age at the initiation of their transition and a clear majority of them express the desire for reproductive potential after transition [29, 30]. This is almost impossible, as irreversible transition means losing the option for having children. Dunne reviewed sterilization requirements for transgender people in Europe and found sterilization as the only possible option in 20 European countries; this means that any chance for biological offspring is lost with this transition [31]. This discrimination deeply undermines the fundamental bioethics law, and societies such as WPATH and the Endocrine Society advocate for counseling and detailed explanation of the consequences of treatment and viable options for fertility preservation. In addition, the possibility of sterility following the use of puberty blockers and cross-sex hormones gives rise to further controversy and ethical dilemmas, as do options of cryopreservation prior to the start of cross-sex hormonal therapy and uterus transplantation for trans-women.

As we have previously mentioned, puberty blockers are considered to be the reversible part of the transition, preventing secondary sex characteristics from developing. However, some authors confirmed that these blockers also have an impact on maturation of germ cells, which could be used for preservation of the biological fertility potential [32]. Individuals on puberty suppression therapy may show an interest in offspring but, at the same time, may not want to pass through the wrong puberty in the gender assigned at birth. Thus, their options for offspring are very limited, since prepubertal cryopreservation is still in the experimental stages [33]. There are other questions as well, including their maturity for making these kinds of decisions and the responsibility of their parents as legal guardians. In the literature, a few authors reported the desire of transgender people to have children and found that about half of both trans-men and trans-women wanted offspring after transition [29, 34].

Cryopreservation of embryos, oocytes, or ovarian tissue is a viable option for trans-men. Some authors recommend cryopreservation just before initiation of hormonal transition due to the possibility that cross-sex hormone therapy might cause amenorrhea or affect follicle growth. In cases where the hormonal transition has already started, they suggest an interruption of hormone treatment for minimum 3 months with a goal to revert any potential therapy-induced effects [35]. These could be very aggravating facts, since other doctors reported that majority of transgender individuals did not want to postpone their transition for these procedures. Interestingly, Wallace et al. noticed that transvaginal ultrasound examination, as a necessary part for cryopreservation of embryos and oocytes, is not always in accordance with individuals' male identity and can lead to distress [36].

Sperm cryopreservation, surgical sperm extraction, and testicular tissue cryopreservation could be offered as possibilities for preserving fertility in trans-women. The issues with hormonal therapy exist in this case, too. De Sutter et al. described additional distress, caused by masturbating in clinical settings or sperm banking as a reminder of their former gender [34].

In some countries, cryopreservation is not technically available to the transgender population and thus cannot be offered during the transition. Despite the fact that cryopreservation is a routine procedure in case of malignant diseases, it still remains a controversial topic in less economically developed countries.

In some countries, like USA, sterilization is not mandatory and trans-men can keep their ovaries and uterus for later pregnancy. They must discontinue cross-sex therapy in this period. Light et al. described transgender pregnancies and challenges that come with this phenomenon [37]. Conversely, pregnancy is still not an option for trans-women. There is hope on the horizon from the first successful uterus transplantation, performed by a gynecology team from Sweden [38]. This is a solution for all women suffering from absolute uterine infertility who want to carry their own children. This procedure brings a new insight for researchers, making the possibility for transplantation in trans-women realistic. The main problems could arise from the different anatomy of the male pelvis, as well as from immunosuppressive therapy.

Fertility, including all the related issues and dilemmas, should be discussed very profoundly and meticulously. Transgender population should be informed about all possibilities, advantages, and drawbacks before any treatment and each option should ultimately be the patient's decision.

## 4. Regret and Revision Surgery

There are various levels of regret after GAS. Definite regret happens when the patient wants to get back to their gender assigned at birth after the GAS is performed. They come to surgeons with the request for the restitution of congenital anatomical features. Regret manifests with a more or less pronounced expression of dissatisfaction and second thoughts about the GAS. After suicide, regret could be considered one of the worst possible complications.

Reasons for regret vary greatly. Inadequate social adaptation, comorbidity with certain psychiatric disorders, poor psychological and psychiatric evaluation, and dissatisfaction with aesthetic or functional outcome of GAS can lead to regret. Researchers have concluded that the presence of the following factors can be associated with a risk of regret: age above 30 years at first surgery, personality disorders, social instability, dissatisfaction with surgical results, and poor support from partner or family [39–41].

In 2016, we published a retrospective analysis of seven patients who underwent reversal surgery after regretting undergoing male-to-female GAS elsewhere [42]. Main reasons for regret in these cases were related to inadequate psychiatric assessment. First stages of transition like the “real-life experience” were mostly skipped, cross-sex hormonal therapy was not carried out properly, and letters of recommendation were written by psychiatrists who lacked experience. Also, main diagnostic criteria for gender dysphoria had been neglected. It is therefore important to avoid situations where inadequately trained or inexperienced psychologists or psychiatrists work with transgender patients without supervision or collaboration with more experienced colleagues. Satisfying postoperative results were achieved in all patients. Reversal surgery significantly enhanced their general well-being.

Each regret occurrence represents a major issue for every expert in the field of transgender medicine. Proper diagnosis and listening to and monitoring our patients are of crucial importance for avoiding these kinds of mistakes [43]. Every physician should be aware that not all individuals suffering from GD want or need all three elements of therapy.

## 5. Conclusion

All physicians included in gender dysphoria treatment are facing great bioethical challenges and dilemmas. A multidisciplinary approach is necessary, but it does not always guarantee a successful outcome. The most sensitive issues are the treatment of transgender youth, fertility and parenting in transgender individuals, and the risk of regret after the irreversible part of the treatment, the gender affirmation surgery. In order to avoid the complex issue of regret, proper preoperative evaluation by experienced professionals, psychologists, and psychiatrists is necessary. More research and studies are necessary to shed light on these issues.
